# Identification of superspreading environment under COVID-19 through human mobility data

**DOI:** 10.1038/s41598-021-84089-w

**Published:** 2021-02-25

**Authors:** Becky P. Y. Loo, Ka Ho Tsoi, Paulina P. Y. Wong, Poh Chin Lai

**Affiliations:** 1 Department of Geography, The University of Hong Kong, Pokfulam Road, Pok Fu Lam, Hong Kong; 2 Institute of Transport Studies, The University of Hong Kong, Pok Fu Lam, Hong Kong; 3 Urban and Transport Research Laboratory, The University of Hong Kong, Pok Fu Lam, Hong Kong; 4 Science Unit, Lingnan University of Hong Kong, Pok Fu Lam, Hong Kong; 5 Centre for Social Policy and Social Change, Lingnan University of Hong Kong, Pok Fu Lam, Hong Kong

**Keywords:** Environmental social sciences, Diseases, Risk factors

## Abstract

COVID-19 reaffirms the vital role of superspreaders in a pandemic. We propose to broaden the research on superspreaders through integrating human mobility data and geographical factors to identify superspreading environment. Six types of popular public facilities were selected: bars, shopping centres, karaoke/cinemas, mega shopping malls, public libraries, and sports centres. A historical dataset on mobility was used to calculate the generalized activity space and space–time prism of individuals during a pre-pandemic period. Analysis of geographic interconnections of public facilities yielded locations by different classes of potential spatial risk. These risk surfaces were weighed and integrated into a “risk map of superspreading environment” (SE-risk map) at the city level. Overall, the proposed method can estimate empirical hot spots of superspreading environment with statistical accuracy. The SE-risk map of Hong Kong can pre-identify areas that overlap with the actual disease clusters of bar-related transmission. Our study presents first-of-its-kind research that combines data on facility location and human mobility to identify superspreading environment. The resultant SE-risk map steers the investigation away from pure human focus to include geographic environment, thereby enabling more differentiated non-pharmaceutical interventions and exit strategies to target some places more than others when complete city lockdown is not practicable.

## Introduction

In medical science, epidemiologists called someone who infected an especially large number of other people *“superspreaders”*^1^. The existence of superspreaders is notable as they can accelerate the rates of new infection in a pandemic. With COVID-19 declared a global pandemic by the World Health Organization (WHO) on March 11, 2020, scientists have closely monitored and continuously updated estimates of the effective reproduction number (Rt), “which represents the mean number of secondary infections that result from a primary case of infection at time t”^2^. Such research is necessary because Rt is an indicator of the transmission potential of a disease. A pandemic signals a declining trend only when Rt is below one. More cases are expected when Rt exceeds one. The higher the value of Rt, the faster the transmission and the more alarming the public health risk. Rt for COVID-19 was estimated to be between two and three in early 2020^3^, but recent research suggests a higher number in the range of 4.4 to 11.7^4^. Yet, there is great inter-personal variability of disease transmission with much higher transmissibility risk of a primary case by a superspreader^5,6^. A superspreader in Wuhan infected 14 healthcare workers that resulted in one death while an individual in Chicago who attended a dinner, a funeral and a birthday party was responsible for 15 new infections^1^. Hence, research on identifying, tracing and treating superspreaders is highly important to pandemic control.

**Figure 1:**
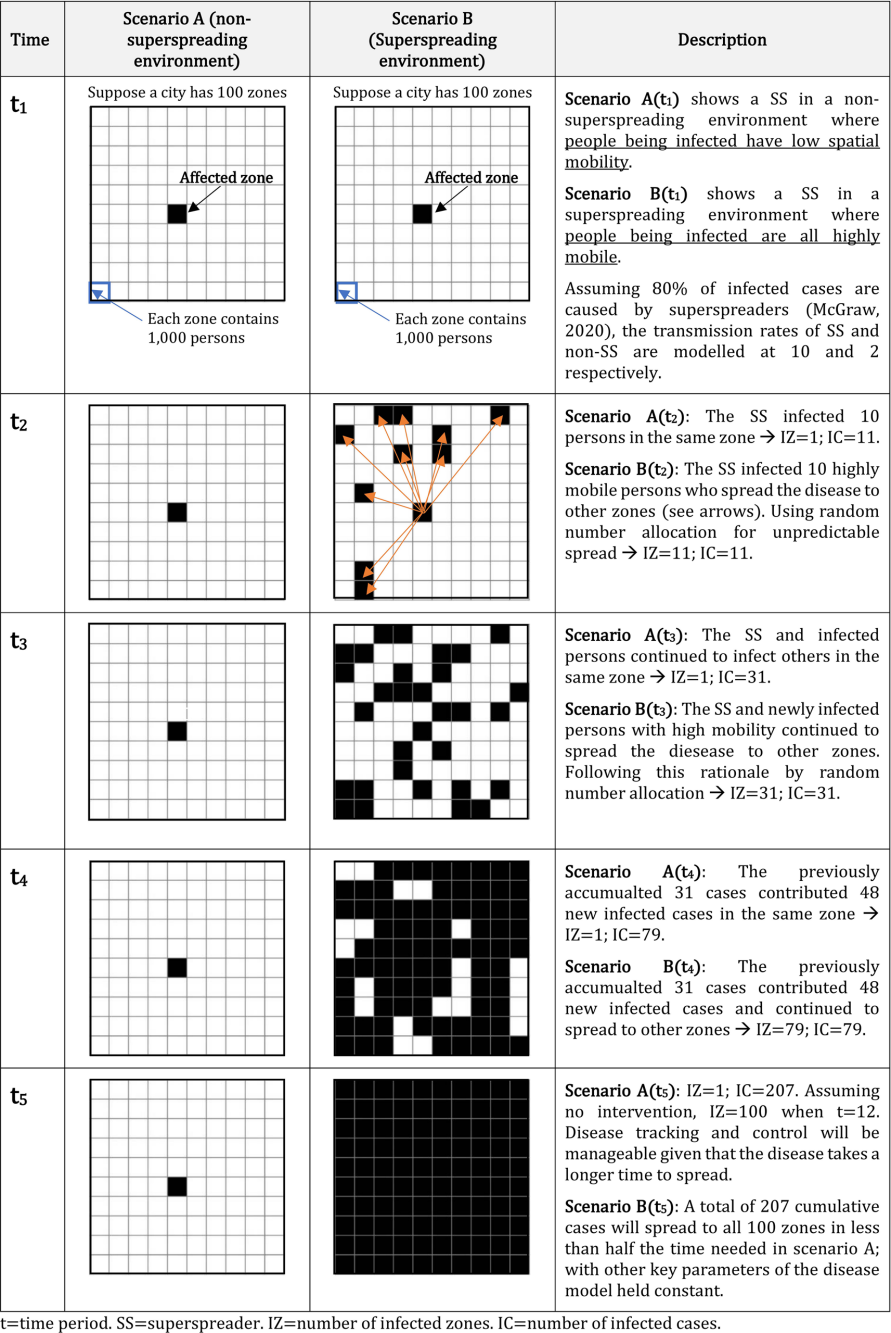
Transmission patterns under the non-superspreading and superspreading environment.

Thus far, the focus of research on superspreaders has primarily been aspatial^1,6,7^. In this paper, we propose to broaden research on superspreaders through geographical modelling of the pandemic spread^8^. Figure 1 is an illustration using a hypothetical example of a city with 100 sub-divisions/zones and with each zone carrying a population of 1,000. Two scenarios of spatial spread could result, assuming that 80% of all infected cases was caused by superspreaders^1^, and the transmission rates of super versus non-super spreaders were modelled at ten and two respectively; where these values may be adjusted according to updated and realistic estimates gathered from field data. Scenario A assumes the presence of a superspreader in a *non-superspreading environment* whereas the superspreader in scenario B exists in a *superspreading environment*. With infectivity yielding similar number of newly infected cases at each time period, the spatial patterns of infection for the two scenarios look very different because of the geographical setting populated by people at two extreme ends of mobility. A non-superspreading environment (Scenario A) is characterised by people at the low end of mobility while a superspreading environment (Scenario B) has highly mobile individuals, including long-distance commuters and those who regularly travel from place to place beyond their local communities.

We argue that a community/district with mobile population must also possess certain amenities or services to turn it into a superspreading environment. This is analogous to Losch modified Christaller’s central place theory^9^, which suggests the locations of retail in urban areas attract people/consumers to purchase goods and services they need (see Supplemental Methods S1.1). Moreover, retail locations in urban versus rural districts exhibit different retail centrality, which is a measure of the retail drawing power or attraction of a given location^10^. The density of streets or transport networks also plays an important role in shaping variations of retail and service intensities within a city^11,12^. Compared with suburban locations, urban cores or central business districts where both high- and low-order retail and transport facilities tend to cluster favour the formation of “attractors” in the settlement layout, exerting a strong pull on consumers living nearby and further away in the region. Consequently, the combined characteristics of higher spatial agglomeration of retail activities and expanse transportation can serve as a proxy for superspreading environment.

Consider the varying spatial patterns of disease transmission under a superspreading environment, we suggest that non-pharmaceutical interventions (NPIs) for disease containment must step beyond the conventional practice of focussing on home and workplace of the infected. Unlike the traditional spatial analysis that associates patients to their home locations^13–15^, this study identifies candidate areas with a heavy concentration of retail and public facilities as potential spreading environments. Our approach aims to predefine high-risk areas of disease spread such that these locations can be targeted to receive more stringent and timely disease prevention and control measures before or during an outbreak. Research on pandemic control have involved scientific studies about the host, pathogen and the environment. Under COVID-19, scientific research on the pathogen (such as the genetic sequences of SARS-CoV-2) has been ongoing at full steam although effective drugs and vaccines to protect human beings are still being tested. It is also noted that a period of at least two days of pre-symptomatic transmission in the community is particularly challenging^2^. Without an effective means of tracking asymptomatic individuals, NPIs are essential components in public health response to the COVID-19 pandemic.

There is recent evidence to suggest that NPIs have been effective in China and beyond^2,16,17^. Specifically, the COVID-19 cases in China could be 67 times higher without NPIs^2,18^. Notwithstanding that these studies have bundled all confinement or lockdown measures as NPIs, they recognized that the effectiveness of different interventions might vary. It is also realised that the economic and social costs of lockdowns can be enormous^19^, as people are confined to homes without being allowed to participate in social, entertainment and leisure activities good for their physical and/or mental health. Knowing that a complete city lockdown incurs enormous economic and social costs, as well as related knock-on effects on physical/mental health and interpersonal violence, it may be beneficial to continue operation of some public facilities deemed important for maintaining people’s daily life and wellbeing. Studies have suggested that permitting limited access to sports centres, libraries, dinning out, and shopping for local residents can be managed with proper disease prevention measures, such as frequent disinfection, mandatory face-masks and physical distancing^20,21^. Under COVID-19, these public facilities have often been targeted for indiscriminate and complete close-down. Referring to NPIs included in the Oxford database^22^, closing of these public facilities may be considered as the intermediate step following school and workplace closures, but before the most drastic measures of stay home order and complete city lockdown.

This paper extends beyond current research that focuses on the pathogen (e.g. characteristics of SARS-CoV-2) or individuals (i.e. the hosts) with a high risk to be superspreaders. It does not focus on biological characteristics of infected persons (e.g. in relation to their immune system) but to examine the environment/space posing different health risks of a transmissible disease. It, therefore, supplements the traditional public health concerns of medical science and microbiology with the spatial dimension of people-environment dynamics. Against this background, this research uses geospatial statistical methods, originated from time geography^23^, to differentiate between exceptionally *“*high-risk superspreading facilities” covering wider spatial catchment areas that attract users from distant locations and those spending substantial periods of time out-of-home, and “local facilities of the same type” but with smaller spatial catchment areas that mainly attract local residents and those spending shorter out-of-home time.

## Methods

This study quantifies and maps potential risk areas of superspreading tendencies in Hong Kong. It aims to illustrate the concept and methods of extracting spatial mobility data in an urban setting to enable the identification of superspreading environments. In the later part of this paper, we also crosschecked the validity of potential risk surface against the patterns of confirmed COVID-19 cases in the city. Two types of human mobility data were extracted from secondary data sources without direct experiments with human participants. Data applied to identify the superspreading environments came from the Travel Characteristics Survey 2011 (TCS-2011), obtained from and approved by the Transport Department of the Hong Kong SAR Government. The TCS-2011 is a territory-wide survey that informs characteristics of trips made by Hong Kong residents on a typical working day. The fieldwork protocols are available in the final report (https://www.td.gov.hk/filemanager/en/content_4652/tcs2011_eng.pdf). The survey contains data of over 35,000 households about their household/personal characteristics and mechanized trip information (e.g. origins/destinations, trip purpose, journey time and transport modes). Data of empirical cases database were publicly available data from the Centre for Health Protection (CHP) of the Hong Kong Government and crowd-sourced information came from local news reports and social media.

We identified six types of retail locations and public facilities where an agglomeration of people and services is likely to take place during a pandemic, viz. bars, shopping centres, karaoke & cinemas, mega shopping malls, public libraries, and sports centres (see Supplemental Methods S1.2). These locations emerged as popular social gathering grounds associated with clusters of locally transmitted cases^24–26^. Locational data for all facilities except bars were readily extracted from the “GeoCommunity Database of Hong Kong”, available from the Survey and Mapping Office of the Lands Department (https://www.landsd.gov.hk/mapping/en/lic/geocom.htm). As “bars” exist in a myriad of combinations (such as cocktail bar, café bar, restaurant-cocktail-bar, pubs, etc.), we searched for any liquor-licensed premises with “bar” in their names (both English and Chinese). Other crowded facilities and services such as places of worship, elderly homes, and restaurants may be chosen but there are operational difficulties due to data availability, privacy concerns, or numerousness of these locations.

We derived human mobility data based on travel-activity diaries from TCS-2011. Although TCS-2011 does not truly reflect movement situations during the pandemic, it is the latest and detailed territorial-wide travel characteristics data for the city. Additional pre-processing of TCS-2011 was necessary (see Supplemental Methods S1.2 & S1.3), as the travel diaries did not differentiate trip purpose for bars and restaurants. Here, we identified street blocks with a high density of bars that also contained three or more trip records in TCS-2011 and used them as a proxy for locating bars in Hong Kong. Bars in neighbouring street block(s) and with TCS trip records were also included for further analysis.

### Space–time concept and measurements

First, we introduce the space–time concept and a method of identifying high-risk public facilities that may give rise to a superspreading environment due not only to the physical locations (e.g. near the central business district) or the nature of facilities (e.g. being bars or shopping centres) but also the characteristics of users (e.g. whether they travel from a wider spatial catchment area and spend substantial out-of-home time with other users having similar lifestyles). Even for the same type of facility like bars, some bars may be more local “watering holes” (corresponding more to scenario A in Fig. 1) while others can attract people from all over the city to hang out and socialize (resembling more to scenario B in Fig. 1).

**Figure 2:**
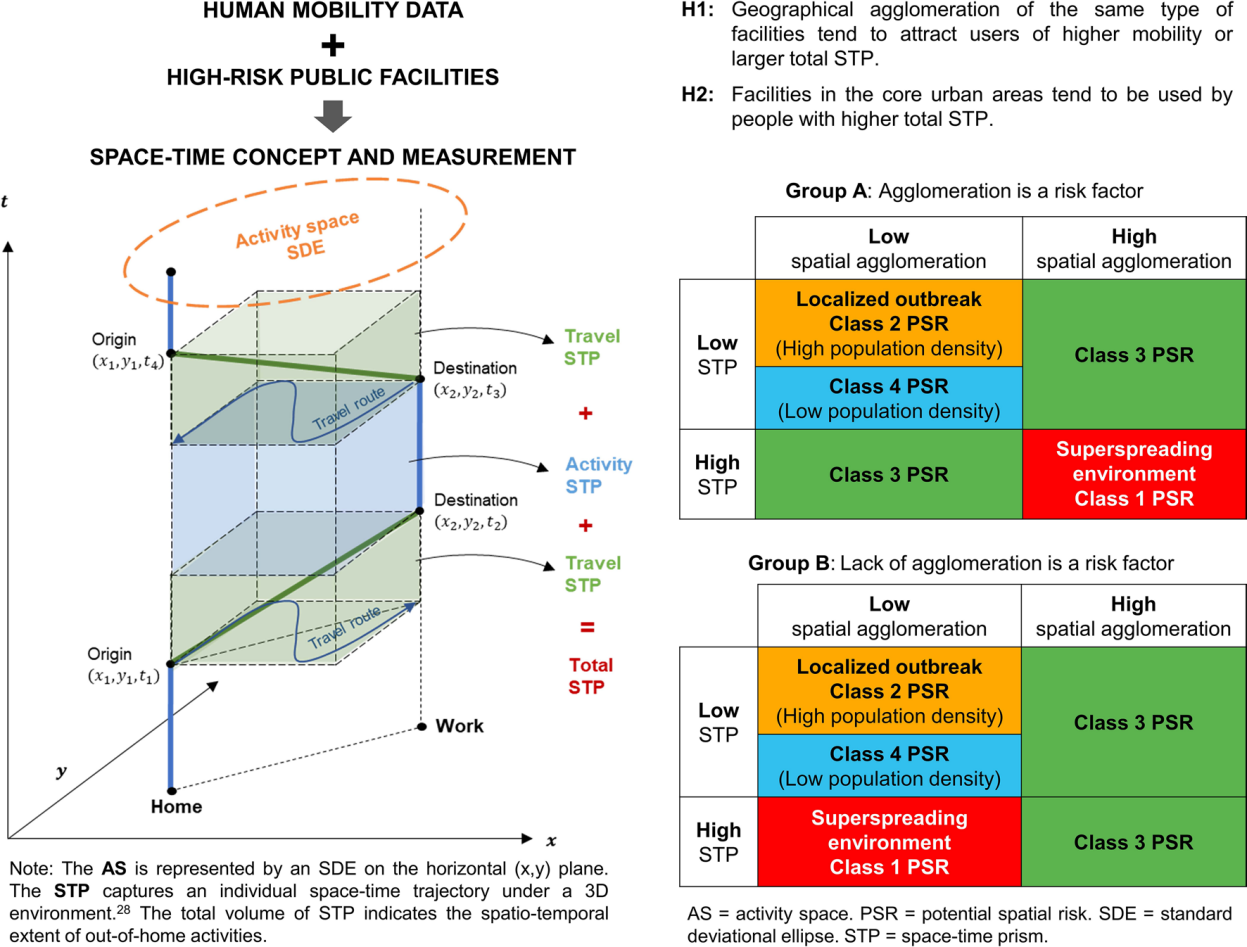
An illustration of the space–time concept and spatial agglomeration.

We made use of time and space information on the mobility pattern of individuals in a city to aggregate the activity space (AS), space–time prism (STP), and the potential path area or potential risk surface for different types of activities (see Supplemental Methods S1.4–S1.6) associated with the selected public facilities^27^. As illustrated in the left panel of Fig. 2, an individual’s recollection of places of visit within a day (including home, all activity locations and transit stops) can be used to delineate a Standard Deviational Ellipse (SDE) which is used as the metric of his/her AS (see Supplemental Methods S1.4). For measuring mobility, we used the 2-dimensional rectangular area bounding the AS and multiply it by the time factor. This measure of STP is a proxy for how far (space) and how long (time) an individual has taken part in out-of-home activities during a day^28^. The AS and corresponding STP for all individuals who visited any of the six types of public facilities recorded in the TCS database were calculated.

### Spatial agglomeration and potential spatial risk (PSR)

We established two hypotheses in relation to the different mobility characteristics of facility users.

H1: Geographical agglomerations of the same type of facilities tend to attract users of higher mobility, as represented by larger total STP.

H2: Facilities in the core urban areas tend to be used by users with higher total STP.

To test the two hypotheses, the geographical agglomeration level (low vs. high) and the geographical locations (urban vs. suburb/new town) of each public facility were analysed. Difference-of-means test was used to see whether the differences of AS and total STP of their respective users were statistically significant at *p* = 0.05.

Based on the six major types of public facilities (i.e. bars, shopping centres, karaoke & cinemas, mega shopping malls, public libraries, and sports centres) and catchment areas of users with a highly mobile lifestyle, we asked whether these combinations could be classified into different classes of “potential spatial risk (PSR)” to differentiate superspreading environments (see right panel of Fig. 2). With spatial agglomeration (high vs. low facility density and urban vs. suburb AS) as the x-axis and users’ total STP (high vs. low) as the y-axis, each facility of the same type was assigned into one of the four quadrants to indicate their PSR classes (see *Group A* and *Group B* tables in the right panel of Fig. 2).

If the above two hypotheses hold true which suggests agglomeration as a risk factor, the high-high quadrant presents the highest PSR in creating a superspreading environment (red quadrant in *Group A*, Fig. 2). However, some locations with few establishments (i.e. a lack of agglomeration or H1 is rejected) that are frequented by highly mobile individuals (i.e. H2 holds true) may also be candidates of a superspreading environment (red quadrant in *Group B*, Fig. 2). Class 2 PSR with high population density (orange sections for both *Groups A & B* in Fig. 2) signals potential locations of localized outbreaks because of low STP. The number of PSR classes will be determined based on the data construct. Our example used two classes (low vs. high) on each dimension to yield four quadrants in total (with the low-low quadrant split by high and low population densities).

To reflect the superspreader characteristics of much higher risks and transmission rates, we weighed the different PSR classes exponentially with a base of ten to standardise and differentiate risk levels. Finally, the risk surface of all six public facilities were integrated to produce a “risk map of superspreading environment” (hereafter referred to as the “SE-risk map”) for the entire city. The method can be adopted in other cities with reasonably well-established data on travel activities and public facilities.

### Tracking AS and validation of the SE-risk map

The CHP hosts a map-centric dashboard showing key information about the pandemic, with daily updates (https://chp-dashboard.geodata.gov.hk/covid-19/en.html). Each case of confirmed and suspected COVID-19 infection gets a unique case number, with the corresponding home location indicated. The CHP and various groups of scientific researchers have tracked infected individuals and identified different “clusters” of COVID-19 cases. At least 40 clusters have been identified as at July 13, 2020 and Supplemental Table S1 lists the ten largest clusters. The top-ranked “bar and music band” cluster not only was the largest so far but also among the earliest in affecting the city.

We chose to focus on these clusters and attempted to obtain more detail about the infected cases from news media and press releases from the government. Many of the recounts of movement and activity by infected individuals were incomplete due to memory lapse and possible editing/word limitations set by local news media. The sequence of visiting different locations was often available from news report but the exact duration of each activity was not available. We could thus compute only the AS (but not total STP) in the validation analysis.

Generally speaking, the wider is the spatial extent of activities of the confirmed cases linked to hot spots identified by the SE-risk map, the higher is the value of the current method to pandemic control and public health. We reconstructed the AS space–time trajectories of the confirmed cases associated with some facilities of class 1 PSR to check the consistency of the findings and establish the value of the SE-risk map in screening facilities based on historical travel-activity records from TCS-2011. To estimate the value of this approach, the empirical patterns of COVID-19 cases or disease clusters associated with these popular public facilities were compiled and the SDE representing AS mapped for geovisualization. Mann–Whitney U test statistics were computed to ascertain the degree of correspondence (*p* = 0.05) between the actual AS of confirmed cases and potential AS based on this approach.

## Results

Table 1 shows the summary results of difference-in-means tests for the six public facilities (see Supplemental Table S2 for detailed statistics). There is evidence to support both H1 and H2 for two types of public facilities: bars and small-medium shopping centers. These public facilities were associated with users with higher AS and STP, especially when they existed in high facility agglomerations and in urban areas (Class 1 PSR in *Group A*, Table 1). H1 is rejected for the other four types of public facilities: karaoke & cinemas, mega shopping malls, public libraries, and sports centres. Our results show that this second group of public facilities was associated with users of higher AS and STP but in areas with lower facility agglomeration (Class 1 PSR in *Group B*, Table 1), perhaps suggesting a lack of similar types of provision in the wider communities. Among the four types of public facilities, H2 is rejected for karaoke and cinemas and sports centres, suggesting that these facilities tended to attract more mobile users in suburbs compared to urban areas. Furthermore, our results show that the remaining public facilities (i.e., public libraries and mega shopping malls) frequented by highly mobile users tended to be the more isolated establishments or locations with low facility agglomeration in the urban setting.

**Table 1:** Results of difference-in-means tests for the six public facilities.

	Activity space (AS)^a^	Space–time prism (STP)*
Group A: Agglomeration is a risk factor		
Bars	**Urban** vs. suburb Low vs. high density	**Urban** vs. suburb Low vs. **high** density
Small-medium shopping centres	**Urban** vs. suburb Low vs. **high** density	**Urban** vs. suburb Low vs. **high** density
Group B: Lack of agglomeration is a risk factor		
Karaoke & cinemas	Urban vs. **suburb** **Low** vs. high density	Urban vs. **suburb** **Low** vs. high density
Mega shopping malls	**Urban** vs. suburb **Low** vs. high density	**Urban** vs. suburb **Low** vs. high density
Public libraries	**Urban** vs. suburb **Low** vs. high density	**Urban** vs. suburb Low vs. high density
Sports centres	Urban vs. **suburb** **Low** vs. high density	Urban vs. **suburb** **Low** vs. high density

^a^Bold letterings indicate groups with a higher mean score (sig. < 0.05).

**Figure 3:**
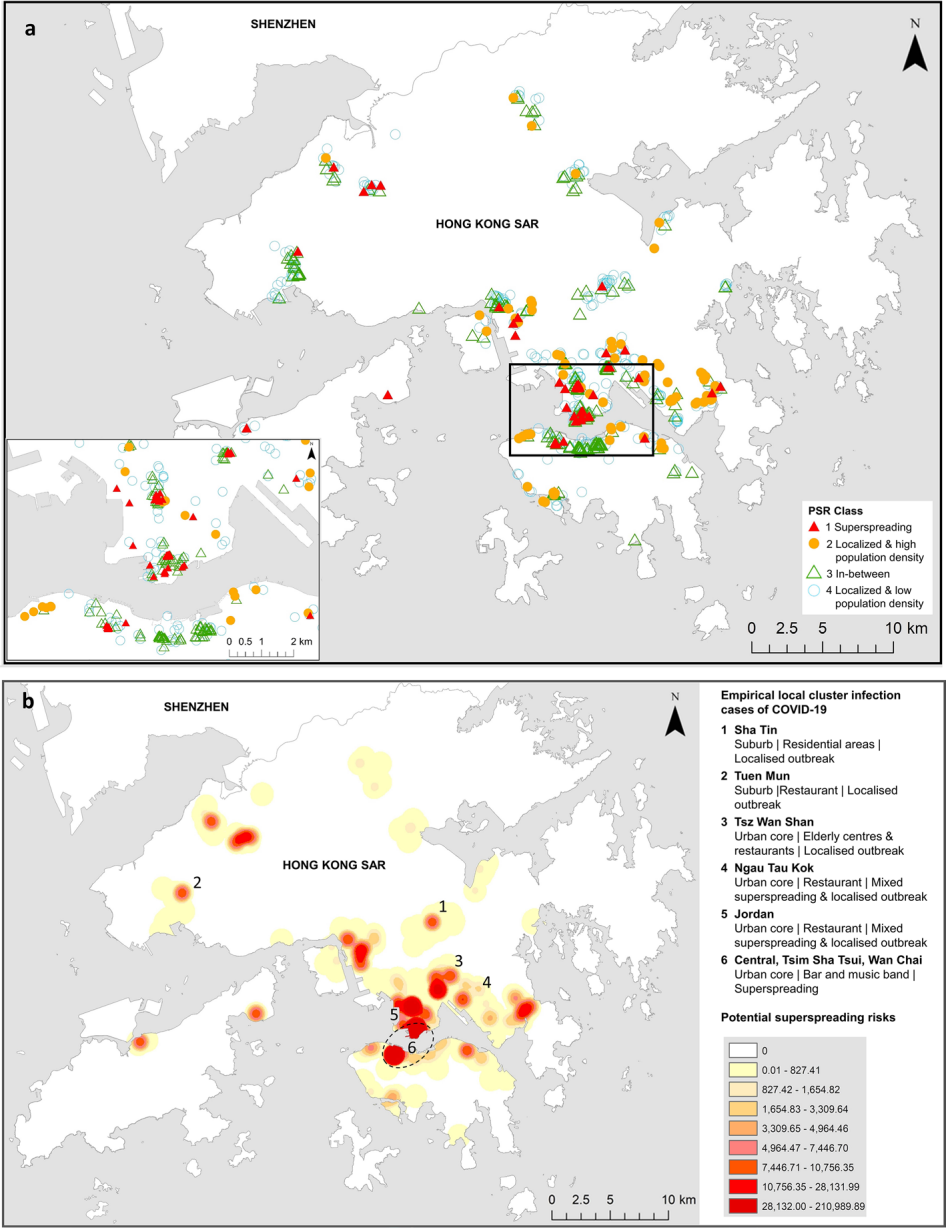
Public facilities by potential spatial risk (PSR) (**a**) and risk map of superspreading environment (SE-risk map) (**b**). (**a**) Symbol locations correspond to four PSR classes as shown in the map legend. (**b**) Top ten locations of empirical local cluster infection (numbered 1–6) plotted against the SE-risk map, a generalized surface of potential superspreading risks with darker shadings indicating higher risks of infection. The empirical local cluster infection cases of COVID-19 shown above included the top 10 infected clusters with the highest number of cases recorded between February and July 2020. (Generated by ArcGIS 10.5, URL: http://www.esri.com/software/arcgis/arcgis-for-desktop).

Next, the full list of six types of public facilities was integrated and the resultant locations assigned into four PSR classes, as shown by *Groups A & B* in Fig. 2. These locations by PSR classes were visualized in Fig. 3a. Of particular interests are the red triangles and orange circles representing areas of superspreading environment and localized infectivity respectively. Figure 3b transformed Fig. 3a from the point symbol representation into a risk surface or the SE-risk map with darker shading denoting higher risks. The numbered locations represent empirical data of actual disease clusters occurring between February and July 2020 inclusive. By visual inspection, the actual disease clusters seem to fall in high-risk areas with identifiable hotspots in Fig. 3b.

**Figure 4:**
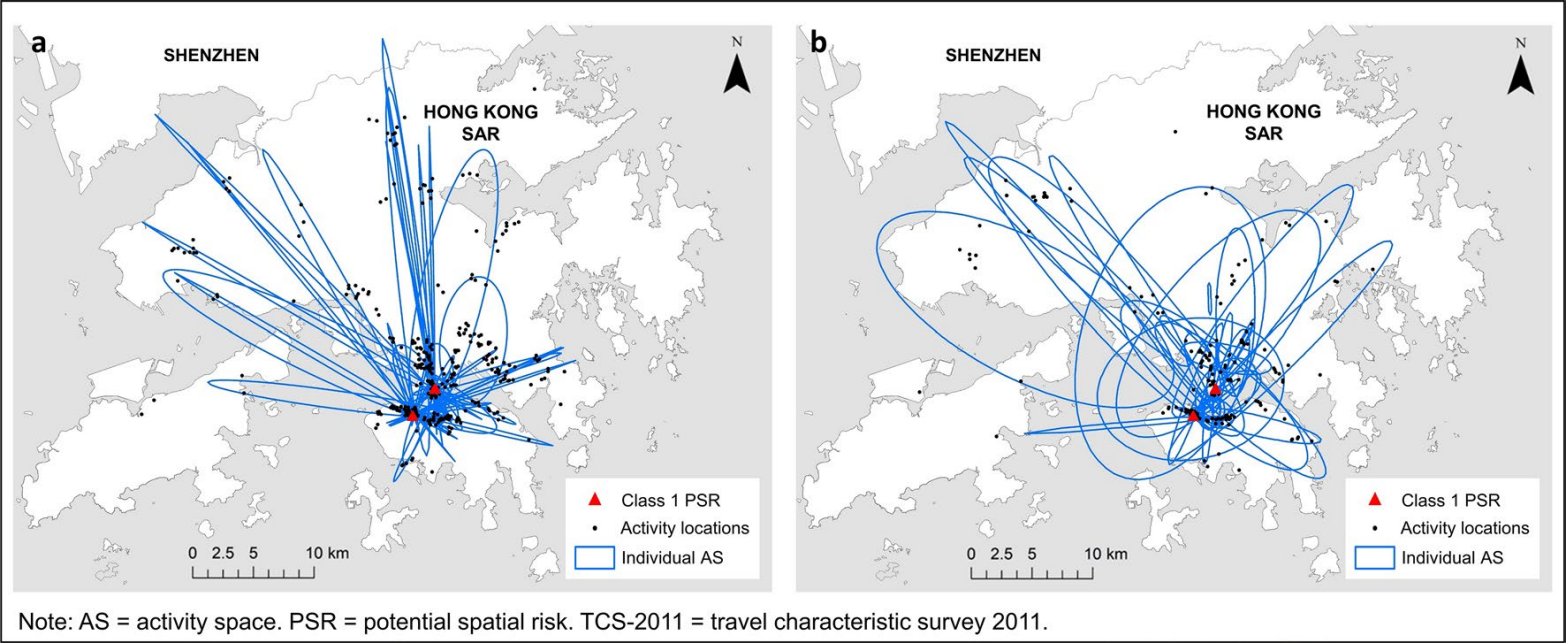
Activity space (AS) based on (**a**) travel characteristic survey versus (**b**) empirical data. No significant difference in the size of AS for both (**a**) and (**b**). Two-sided Mann–Whitney U test indicates that the AS of individuals who visited street blocks of bars in class 1 PSR (*n* = 2,463; mean rank = 1,248.97) and the empirically infected cases from bars (*n* = 35; mean rank = 1,286.74) in class 1 PSR were NOT significantly different in size (*p* = 0.758). Extreme outliers were removed. (**a**) AS of individuals who visited bars in class 1 PSR based on TCS-2011. Two-sided Mann–Whitney U test indicates the AS of individuals visiting bars in class 1 PSR (*n* = 2463; mean rank = 3437.18) is higher than those visiting bars in class 3 PSR (*n* = 3887; mean rank = 3009.68) (*Z* = − 9.06; *p* = 0.000). Extreme outliers were removed. (**b**) AS of individuals infected with COVID-19 and had visited bars in class 1 PSR. Two-sided Mann–Whitney U test indicated the AS of individuals visiting bars in class 1 PSR (*n* = 35; mean rank = 23.77) is higher than those visiting bars in class 3 PSR (*n* = 8; mean rank = 14.25) (*Z* = − 1.94; *p* = 0.026). Extreme outliers were removed. (Generated by ArcGIS 10.5, URL: http://www.esri.com/software/arcgis/arcgis-for-desktop).

To consider the empirical value of the analysis to inform policy makers, we identified two bars designated as class 1 PSR by our method and plotted the AS space–time trajectory based on TCS-2011 (Fig. 4a). Our method shows AS of wide coverage indicating patrons of bars were not limited to nearby residents. Then, we reconstructed the AS time–space trajectory of the infected cases from the same bars based on information from tracing studies conducted by the CHP and news reports from local media (Fig. 4b). The derived AS show a large spatial extent of comparable pattern. Mann–Whitney U test indicates no significant difference in the areal coverage of AS between our method and the actual occurrence (*p* = 0.76).

## Discussion

Targeting the environment to complement superspreader research is needed. Our study illustrates the potential value of mapping hot spots of superspreading environment in an ex-ante manner by combining geographical settings with pre-pandemic human mobility data. The SE-risk map can serve as a useful reference for policy makers in targeting facilities for more differentiated NPIs. The wisdom of Dr John Snow more than 150 years ago has highlighted the importance of associating disease cases (that is, cholera) with the location of public facilities (that is, public water pumps)^29^. Given the challenge of a pandemic with no effective vaccine and cures, NPIs are necessary to “avoid peaks of cases that would overwhelm hospital and intensive care unit (ICU) capacity and result in hundreds of thousands of deaths”^30^^(p 1)^.

Current observations and the global trend have indicated that the COVID-19 pandemic is likely to last for some time. It has become increasingly difficult to enforce citywide lockdowns that carry serious political, economic and social (including health) consequences. Identifying individuals with the disease (at the pre-symptomatic stage), reducing the time for diagnosis and laboratory tests, proper isolation of the infected, and tracing of the whereabouts of the individuals that they have been in contact with, will remain critical in this fight against coronavirus. These persistent efforts have put pressure on people’s everyday life, and the already strained healthcare and laboratory resources worldwide. One major challenge in a pandemic is the rapid and unpredictable transmission of disease associated with superspreaders. Using the conceptual and methodological framework of superspreading environment, our analysis has demonstrated the potential value of integrating geographical knowledge and human mobility patterns to predict the “origins” of disease clusters based on the activity type, facility features, and users’ travel-activity pattern. One limitation of the study is that we had to use travel characteristics survey data from 2011 to analyze a pandemic of 2020. Since that year, complex changes in people’s activity-travel patterns may have happened as the city’s urban form and transport system evolved. This issue may hinder the accuracy of the study. With more updated data, the results of the study would be more accurate. Despite this limitation, the usefulness of the technique was validated. Besides, the selection of public facilities conducive to disease spread and insights derived from the potential risk surface may be more applicable to COVID-19 in particular and other respiratory pathogens in general, and somewhat relevant to infectious diseases with other modes of transmission. The generalizability of the potential risk surface may thus be constrained.

In terms of future research directions, more factors which are known to contribute to the formation of superspreading environment, such as poor indoor ventilation, can be considered and integrated into the current analysis. In parallel, there is a need to refine the research by considering more characteristics both about the users and the facilities. For the former, superspreaders of certain occupations working in a superspreading environment may result in even higher public health risk. For the latter, facilities located at mixed commercial-residential areas can be an extra risk factor. In addition, our current study made use of historical travel characteristic data to compute AS and STP of individuals. The widespread use of smart cards and mobile devices with self-tracking applications and big data analytic software can provide more refined mobility data to construct the SE-risk map using our proposed method^31,32^. Nonetheless, NPIs that focus on modified human behaviours from wearing masks and hand disinfection to reducing travel and social activities must go hand in hand in fighting the pandemic.

## Supplementary information

Supplementary information.

## Data Availability

All data generated during this study are included in the main text and supplementary information. The mobility data from Travel Characteristics Survey 2011 (TCS-2011) used to support the findings in this study are not publicly available because the dataset is under a license from the data provider. Some aggregated information is publicly available online (https://www.td.gov.hk/filemanager/en/content_4652/tcs2011_eng.pdf). The mobility data of empirical cases were compiled by the authors based on publicly available data from the Centre for Health Protection of the Hong Kong Government (https://www.coronavirus.gov.hk/ eng/index.html) and crowd-sourced information from local news reports and social media.
