# Supplementary material for: Identification of superspreading environment under COVID-19 through human mobility data
Source: Sci Rep. 2021 Feb 25;11:4699. doi: 10.1038/s41598-021-84089-w (PMC7907097; doi:10.1038/s41598-021-84089-w)
Supplement: Supplementary file 1 — Supplementary information. [file 41598_2021_84089_MOESM1_ESM.docx]

**Supplementary Information:**

**Title**

Identification of superspreading environment under COVID-19 through human mobility data

**Authors**

Becky P.Y. Loo ^1,2,3^, Ka Ho Tsoi ^1,3^, Paulina P.Y. Wong ^4,5^, Poh Chin Lai ^1,2^ *

^1^ Department of Geography, The University of Hong Kong, Hong Kong

^2^ Institute of Transport Studies, The University of Hong Kong, Hong Kong

^3^ Urban and Transport Research Laboratory, The University of Hong Kong, Hong Kong

^4^ Science Unit, Lingnan University of Hong Kong, Hong Kong

^5^ Centre for Social Policy and Social Change, Lingnan University of Hong Kong, Hong Kong

* Correspondence to:

Poh Chin Lai, Ph.D.

Department of Geography

The University of Hong Kong

Pokfulam Road, Hong Kong

Tel: (852) 3917-2830

E-mail: pclai@hku.hk

# **Supplemental Methods**

## **S1.1 Christaller’s Central Place theory**

The assumptions of the original theory (including even terrain, evenly distributed population, evenly distributed resources, similar purchasing power, preference for nearest market, equal transportation cost, and perfect competition) are not considered here.

## **S1.2 Types of public facilities**

The public facilities included bars, karaoke and cinemas, public libraries, sports centres, small and medium shopping centres (≤240 shops), and mega shopping malls (>240 shops). Shopping malls were originally one category but a more in-depth analysis revealed that potential risk characteristics of mega malls environment were very different from smaller shopping centres; hence the two distinct groups.

Except for bars, all other five types of facilities were mapped by their geocoded locations registered in the GeoCommunity Database of Hong Kong. Some additional pre-processing steps were required for bars, including restaurants serving alcoholic beverages, which often are not clearly distinguished in Hong Kong. The steps outlined below can also be applied to other public facilities lacking comprehensive spatial information.

***Step 1*:** Identify the locations of bars based on names of business establishments (with license to sell alcoholic drinks) that contain “bars” in both English and Chinese characters. Also, identify the locations of major bars through recognised sources, such as the Bar Map published by the Hong Kong Bar & Club Association.

***Step 2*:** Calculate the bar density in each street block.

***Step 3*:** Identify the street block of the highest bar density in each district. To ensure sufficient records for subsequent data analysis, the street block must contain >3 trip records for restaurant/bar purpose in TCS-2011.

***Step 4*:** Bars in adjacent and neighbouring street block(s) with trip records will also be geocoded and included in the analysis.

## **S1.3 Pre-processing steps for TCS-2011**

***Step 1: Sample identification and data extraction from TCS-2011***

All records in TCS-2011 are anonymous without personal identifier. Anonymized individuals with at least one visit to public facilities (specified above in Supplemental Methods S1.2) during the survey day were identified for further analysis. Their travel data - including trip origin, trip destination, departure time, arrival time and transport mode - were extracted from TCS-2011 whilst travel time and activity duration computed from the extracted data.

***Step 2: Transforming raw data into spatiotemporal coordinates***

This step was conducted in the ArcGIS environment (e.g. ArcMap 10.5 or ArcGIS Pro). It should be noted that the locational information in TCS-2011 was recorded at the street block/village cluster (SBVC) level, which is the smallest census unit in Hong Kong. The centroid (*x*,*y*) of a SBVC registers its spatial location. The time variable (*t*) was calculated based on the arrival and departure time of the trip(s) made by an individual on the survey day. Hence, the spatiotemporal information of an individual can be complied (see Table S1.1). The “Display XY Data” function in ArcMap can facilitate 3D geometry and store the *z*-field (e.g. elevation) to visualise the data. In our case, the z-field is the time dimension (*t*). The row entries of *t*=0 and *t*=1 in spatiotemporal information of Table S1.1 illustrate the beginning and the ending of trip data for the day. With these coordinate data, the spatiotemporal movements can be mapped in a 3D environment.

**Table S1.1: An illustration of transforming raw data into spatiotemporal information**

**Raw data in TCS-2011**

| **lD** | **Trip** | **Origin (SBVC)** | **Destination (SBVC)** | **Departure time** | **Arrival time** | **Transport mode** | **Trip purpose** |
| --- | --- | --- | --- | --- | --- | --- | --- |
| 1 | 1 | 476324 | 862790 | 08:00 | 08:45 | Rail | Shopping |
| 1 | 2 | 862790 | 476324 | 18:00 | 19:00 | Bus | Home |
| … | … | … | … | … | … | … | … |

**Spatiotemporal information**

| **Individual** | **Trip** | ***x*** | ***y*** | ***t*** | **Place** |
| --- | --- | --- | --- | --- | --- |
| 1 | - | 845046.4 | 818798.5 | 0 | Home |
| 1 | 1 | 845046.4 | 818798.5 | 0.33 | Home |
| 1 | 1 | 835746.1 | 817008.2 | 0.36 | Shopping |
| 1 | 2 | 835746.1 | 817008.2 | 0.75 | Shopping |
| 1 | 2 | 845046.4 | 818798.5 | 0.79 | Home |
| 1 | - | 845046.4 | 818798.5 | 1 | Home |

Note: The figures are for illustrative purpose and not real.

***Step 3: Three-dimensional visualisation of space-time trajectories***

This step was conducted in the ArcGIS environment. Having consolidated all the spatiotemporal coordinates (points) for each individual on the survey day, we connected the points using the “Points to Line” function to visualise the 3D space-time trajectory. Time-space trajectories have been a useful methodology to examine individual travel and activity patterns, time-budget constraints and exposure to surrounding environments^1,2^. The 3D space-time paths can be shown in ArcScene. A sloping line represents travel between locations (change of both time and spatial location) whereas a vertical line represents staying at the same place (change of time only). This step focusses on visualisation only. Advanced steps of measuring space-time trajectories based on travel routes and other information are discussed under Supplemental Methods S1.5.

## **S1.4 Activity space (AS)**

An activity space (AS) is a proxy measure to reflect the geographical extent within which a person travels or moves about during his/her course of activities to meet individual needs and desires^3,4^. In other words, an AS captures places frequented by an individual to perform daily activities, and a potential AS is indicated by the potential path area^5^. There are several common methods to operationalise AS, such as the standard deviational ellipse (SDE), path buffer area and convex polygons^6^. This study adopts the SDE approach, which is a Euclidean measure. The detailed methodology and calculations of SDE in GIS are available at <https://pro.arcgis.com/en/pro-app/tool-reference/spatial-statistics/h-how-directional-distribution-standard-deviationa.htm>. Based on the spatial coordinates of locations of visit (Supplemental Methods S1.3), the AS of an individual was constructed and calculated in the ArcGIS environment.

## **S1.5 Space-time prism (STP)**

A space-time prism (STP) illustrates the spatiotemporal extent of an individual’s travel and daily activities. Unlike the traditional potential path area where an individual can travel given the time budget constraint^7^, this study uses the STP as a proxy to indicate the spatiotemporal extent of an individual’s travel movement and out-of-travel-activities. The major difference between the AS and STP approaches is that STP integrates the time dimension to capture the duration of various activities. This notion is contingent upon the fundamental concept of time geography that space and time are inseparable. Hence, STP indicates that an individual situated at a particular location at a specific time will have a set of spatiotemporal coordinates (*x*, *y*, *t*). The change of *x*, *y* and *t* along the travel trajectory will indicate how an individual moves across a space-time environment.

The minimum rectangular area bounding the origin and destination with the travel route represents the overall space incurred in travel (Figure 2 in the main text). The height dimension of *t* indicates the actual duration of travel (i.e. the difference between departure time at the origin and arrival time at the destination). The product of these two components thus produces a space-time volume that can be visualised as a STP. Three additional steps are involved in the calculation of the size of STP, as described below.

***Step 1: Construction of shortest paths***

We employed the “Network Analyst” extension in ArcMap 10.5 to generate the shortest paths in order to delineate the two-dimensional space in a STP that encompasses travel routes of an individual. As TCS-2011 collects data on origins and destinations without details of the travel routes, the travel route between an origin and a destination was estimated based on the shortest path of the respective transport modes. For instance, the shortest path of an individual travelling by rail was generated along the rail network. In the case of multimodal trips, routes for all trip legs were derived separately.

***Step 2: Minimum bounding geometry***

To measure areas an individual traversed along the travel routes, we used a proxy of the minimum rectangular area enclosed by the origin and the destination. We employed the “Minimum Bounding Geometry” function in ArcMap 10.5 and specified “Rectangle by Area” to generate the bounding area regardless of the orientation.

***Step 3: Calculation of the volume***

The volume of a STP is the product of the minimum bounding area and travel time/activity duration.

**S1.6 Potential risk surface**

The potential risk surface indicates the potential risks of transmission arising from the superspreading environment. Following the discussion and typology developed in Figure 2 in the main text, the six types of facilities were integrated and categorised by the four classes of potential spatial risk (PSR). The spatial datasets of six public facilities were merged into one data file to generate the map of potential risk surface shown in Figure 3a (in main text).

# **Supplemental Results**

**Table S2.1: Local cluster infection cases of COVID-19 in Hong Kong, February to July 2020. Table lists top ten clusters by total number of infected cases.**

| **Rank** | **Group or cluster name** | **Duration** | **Cases** |
| --- | --- | --- | --- |
| 1 | Bar and music band | 18/3/2020 - 13/4/2020 | 103 |
| 2 | Kong Tai Care for the Aged Centre Ltd (Tsz Wan Shan) | 7/7/2020 – 17/7/2020 | 44 |
| 3 | Sun Fat Restaurant (Jordan), Bun Kee Congee & Noodle (Ping Shek Estate) and Kin Wing Canteen (Tuen Mun) | 5/7/2020 – 17/7/2020 | 39 |
| 4 | Green River Restaurant (Tsz Wan Shan) | 11/7/2020 – 17/7/2020 | 22 |
| 5 | Buddhist temple in North Point | 19/2/2020 8/3/2020 | 19 |
| 6 | Wedding banquet in Discovery Bay | 18/3/2020 -24/3/2020 | 15 |
| 7 | Windsor Restaurant (Tsz Wan Shan) | 30/3/2020 – 17/7/2020 | 15 |
| 8 | Hot-pot dinner | 9/2/2020 - 12/2/2020 | 13 |
| 9 | Luk Cheun House (Shatin) | 31/5/2020 – 13/6/2020 | 12 |
| 10 | Ming Chuen House of Shui Chuen O Estate (Shatin) | 7/7/2020 – 10/7/2020 | 12 |

Source: Authors

## **Table S2.2: Results of difference-in-means tests for AS and STP of the six public facilities. Bold letterings indicate groups with a higher mean score (sig.<0.05).**

**A. Activity space (AS)**

|  | **N** | | **Mean** | **t** | **Sig.** |
| --- | --- | --- | --- | --- | --- |
| **Bars** | | | | | |
| Urban | 8,110 | | **14.35** | 5.76 | **0.000** |
| Suburb | 4,598 | | 12.00 |  |  |
| Low density | 9,539 | | 13.29 | -1.79 | 0.073 |
| High density | 3,170 | | **14.13** |  |  |
| **Small-medium shopping centres** | | | | | |
| Urban | | 99,436 | **8.45** | 33.29 | **0.000** |
| Suburb | | 83,076 | 6.10 |  |  |
| Low density | | 135,867 | 6.77 | -25.85 | **0.000** |
| High density | | 46,645 | **9.17** |  |  |
| **Karaoke & cinemas** | | | | | |
| Urban | 4,101 | | 11.78 | -7.68 | **0.000** |
| Suburb | 590 | | **18.97** |  |  |
| Low density | 3,416 | | **15.57** | 22.83 | **0.000** |
| High density | 1,275 | | 4.97 |  |  |
| **Mega shopping malls** | | | | | |
| Urban | 27,426 | | **5.99** | 11.18 | **0.000** |
| Suburb | 30,023 | | 4.89 |  |  |
| Low density | 44,298 | | **5.55** | 4.79 | **0.000** |
| High density | 13,152 | | 4.98 |  |  |
| **Public libraries** | | | | | |
| Urban | 2,692 | | **7.95** | 15.08 | **0.000** |
| Suburb | 1,600 | | 3.76 |  |  |
| Low density | 3,963 | | **6.51** | 3.50 | **0.001** |
| High density | 329 | | 5.01 |  |  |
| **Sports centres** | | | | | |
| Urban | 4,363 | | 5.84 | -4.72 | **0.000** |
| Suburb | 1,719 | | **8.34** |  |  |
| Low density | 4,645 | | **7.05** | 8.04 | **0.000** |
| High density | 1,437 | | 4.92 |  |  |

**B. Space-time prisms (STP)**

|  | **N** | **Mean** | **t** | **Sig.** |
| --- | --- | --- | --- | --- |
| **Bars** | | | | |
| Urban | 8,110 | **223.33** | 10.32 | **0.000** |
| Suburb | 4,598 | 151.34 |  |  |
| Low density | 9,539 | 171.03 | -9.98 | **0.000** |
| High density | 3,170 | **276.28** |  |  |
| **Small-medium shopping centres** | | | | |
| Urban | 99,436 | **102.16** | 19.06 | **0.000** |
| Suburb | 83,076 | 86.53 |  |  |
| Low density | 135,867 | 89.53 | -22.08 | **0.000** |
| High density | 46,645 | **111.11** |  |  |
| **Karaoke & cinemas** | | | | |
| Urban | 4,101 | 172.81 | -12.47 | **0.000** |
| Suburb | 590 | **343.95** |  |  |
| Low density | 3,416 | **253.31** | 23.38 | **0.000** |
| High density | 1,275 | 84.55 |  |  |
| **Mega shopping malls** | | | | |
| Urban | 27,426 | **88.27** | 7.84 | **0.000** |
| Suburb | 30,023 | 78.32 |  |  |
| Low density | 44,298 | **89.18** | 17.36 | **0.000** |
| High density | 13,152 | 62.48 |  |  |
| **Public libraries** | | | | |
| Urban | 2,692 | **84.90** | 2.60 | 0.009 |
| Suburb | 1,600 | 72.41 |  |  |
| Low density | 3,963 | **80.85** | 1.71 | 0.088 |
| High density | 329 | 72.99 |  |  |
| **Sports centres** | | | | |
| Urban | 4,363 | 63.86 | -4.82 | **0.000** |
| Suburb | 1,719 | **83.84** |  |  |
| Low density | 4,645 | **71.44** | 2.96 | **0.003** |
| High density | 1,437 | 63.23 |  |  |

**References:**

1. Lam, W.W.Y., Yao, S. & Loo, B.P.Y. Pedestrian exposure measures: A time-space framework. *Travel Behav Soc*. **1**(1), 22–30 (2014).
2. Lam, W.W.Y., Loo, B.P.Y. & Yao, S. Towards exposure-based time-space pedestrian crash analysis in facing the challenges of ageing societies in Asia. *Asian Geogr*. **30**(2), 105–125 (2013).
3. Miller, H. J. A measurement theory for time geography. *Geogr. Anal*. **37**(1), 17–45 (2005).
4. Gesler, W.M. & Albert, D.P. How spatial analysis can be used in medical geography. *Spatial Analysis, GIS and Remote Sensing Applications in the Health Sciences*. (ed. Albert, D.P., Gesler, W.M. & Levergood, B.) 11–38. (Chelsea, MI, Ann Arbor Press, 2000).
5. Loo, B.P.Y. & Lam, W.W.Y. Does neighbourhood count in affecting children's journeys to schools?
   *Child Geogr*. **13**(1), 89–113 (2015).
6. Hirsch, J.A., Winters, M., Clarke, P. & McKay, H. Generating GPS activity spaces that shed light upon the mobility habits of older adults: a descriptive analysis. *Int. J. Health Geogr*. **13**(1), 51 (2014).
7. Hägerstrand, T. What about People in Regional Science? *Papers of the Regional Science Association*. **24**, 1–12 (1970).
